# Food Bioactive Compounds against Diseases of the 21st Century 2016

**DOI:** 10.1155/2017/1750823

**Published:** 2017-02-05

**Authors:** Blanca Hernández-Ledesma, Chia-Chien Hsieh, Cristina Martínez-Villaluenga

**Affiliations:** ^1^Instituto de Investigación en Ciencias de la Alimentación CIAL, CSIC-UAM, CEI UAM-CSIC, Nicolás Cabrera 9, 28049 Madrid, Spain; ^2^Department of Human Development and Family Studies (Nutritional Science & Education), National Taiwan Normal University, No. 162, Heping East Road, Section 1, Taipei, Taiwan; ^3^Institute of Food Science, Technology and Nutrition (ICTAN-CSIC), Juan de la Cierva 3, 28006 Madrid, Spain

Nowadays, obesity, metabolic syndrome, and other chronic diseases including cardiovascular disease, stroke, diabetes, cancers, immune disorders, and chronic respiratory disease are becoming the leading causes of global morbidity and mortality. Diet pattern and physical exercise have been found to be the most important factors impacting on these chronic diseases. Because they are preventable, modifications in diet and lifestyle habits are currently recognized as promising strategies to prevent and/or treat these diseases. In addition to their content in essential nutrients, foods contain a wide range of bioactive compounds. In the last years, the number of studies evaluating the physiological activities of food-derived bioactives has markedly increased. Cell culture, animal models, and human trials are being performed to demonstrate the benefits of foods on health and to identify and characterize the responsible compounds. Therefore, the present special issue summarizes the most recent advances on bioactive compounds derived from food making them to be recognized as promising strategies in prevention, auxiliary therapy, or even cure of different chronic diseases of the 21st century. The selected papers represent a novel and many-facet knowledge, which we have the pleasure of sharing with the readers.

In a paper entitled “Delphinidin-Rich Maqui Berry Extract (Delphinol®) Lowers Fasting and Postprandial Glycemia and Insulinemia in Prediabetic Individuals during Oral Glucose Tolerance Tests,” J. L. Alvarado et al. investigate the effect of a delphinidin-rich extract obtained from maqui berry and known as Delphinol on glucose metabolism in prediabetic humans challenged with pure glucose. These authors demonstrate, for the first time, the ability of Delphinol to simultaneously reduce fasting blood glucose and insulin levels in patients when administered in a single dose. These effects are suggested to be mediated through multiple mechanisms including inhibition of intestinal glucose transporters, an incretin-modulating effect on insulin secretion, and improvement of the insulin sensitivity in target tissues.

In the last years, the number of patients suffering of neuropathic pain, defined as pain caused by a lesion or disease of the somatosensory nervous system, has increased rapidly. However, to date, drugs used to alleviate this pain are not completely effective and provokes undesired side effects, such as tolerance and physical dependence. In the paper entitled* “*Food-Derived Natural Compounds for Pain Relief in Neuropathic Pain,” E. Y. Lim and Y. T. Kim summarize the processes implicated in the etiology and progress of neuropathic pain and the potential benefits of C-C motif chemokine receptor 2 (CCR2) antagonists for treatment of this chronic disorder. In addition, plant food-derived compounds that have demonstrated in animal models to be useful for neuropathic pain alleviation are described in detail.

Skeletal muscle injury is a common clinical issue that can be caused by several conditions including direct trauma, prolonged training, ischemia, or myotoxins. Food-derived peptides and amino acids may function as anti-inflammatory agents as illustrated in the original research work entitled “Shrimp Protein Hydrolysate Modulates the Timing of Proinflammatory Macrophages in Bupivacaine-Injured Skeletal Muscles in Rats” by J. Dort et al. This study provides information on the* in vivo* effects of shrimp protein hydrolysate consumption on chemically injured rat skeletal muscle. Protein hydrolysate feeding improved resolution of inflammation in skeletal muscles through modulation of proinflammatory macrophages accumulation that can induce a generalized beneficial effect on muscle regeneration.

Skin suffers inappropriate ultraviolet (UV) exposure causing dermal photo-damage and photo-aging, resulting in partial inflammation, redness, swelling, and tissue damage. In a paper entitled “Djulis (*Chenopodium formosanum* Koidz.) Water Extract and Its Bioactive Components Ameliorate Dermal Damage in UVB-Irradiated Skin Models,” Y.-H. Hong and coworkers pointed that Djulis treatment protected skin HaCaT cells against UV-induced inflammation, oxidative stress, and increased the cell viability. By an* in vivo* study, it was also demonstrated that administration of the extract exerted protection against UV challenge in skin of mice. In addition, the authors found that the contributive compounds of* Djulis* are mainly rutin and chlorogenic acid.

The rising challenge of bacterial resistance to antibiotics has promoted the need for the development of natural products with antibacterial and antioxidant activity to effectively manage many infectious diseases. The study “Antibacterial and Antioxidant Properties of the Leaves and Stem Essential Oils of* Jatropha gossypifolia* L.,” by S. O. Okoh et al. proposes the use of leaves and stem essential oils of the medicinal plant* Jatropha gossypifolia* as a potential therapeutic agent for the treatment of microbial infections. This study provided a detailed characterization of the fatty acid composition of essential oils and described their antiradical and antibacterial properties indicating their potential therapeutic uses in the prevention or treatment of infectious diseases.

In a paper entitled “DHA and EPA Content and Fatty Acid Profile of 39 Food Fishes from India,” B. P. Mohanty and coworkers demonstrated that fish is an important source of polyunsaturated fatty acids (PUFA) and has a unique advantage. Docosahexaenoic acid (DHA) and eicosapentaenoic acid (EPA) are present in high concentration in sea creatures with high fat composition and play important roles in fetal brain development, lipid metabolism, cognitive support, preventing atherosclerosis, dementia, and immune disorders. This article reported that 39 fish species from India, especially* Tenualosa ilisha*,* Sardinella longiceps*,* Nemopterus japonicus*, and* Anabas testudineus*, are rich sources of DHA and EPA. Promotion of these species as DHA fish sources would enhance their utility in public health nutrition.

Hypoglycemic effect of the main antidiabetic compound in mulberry latex, the glucose analogue 1-deoxynojirimycin, is well-known to be due to inhibition of intestinal *α*-glucosidase. Further understanding of 1-deoxynojirimycin glucose lowering effects were examined in the study* “*An Evaluation of 1-Deoxynojirimycin Oral Administration in Eri Silkworm through Fat Body Metabolomics Based on 1H Nuclear Magnetic Resonance” by C. Wen et al. NMR-based metabonomics analysis of the Eri silkworms fat body indicated that 1-deoxynojirimycin has a positive impact on the reverse energy metabolism. These results reinforce the evidence for the therapeutic application of 1-deoxynojirimycin as food supplement, ingredient, or nutraceutical in diabetes.

In a paper entitled “Roe Protein Hydrolysates of Giant Grouper (*Epinephelus lanceolatus*) Inhibit Cell Proliferation of Oral Cancer Cells Involving Apoptosis and Oxidative Stress,” J.-I. Yang et al. evaluated the impact of ultrafiltrated roe hydrolyzates obtained from giant grouper* (Epinephelus lanceolatus)* on proliferation of oral cancer Ca9-22 and CAL 27 cells. In addition, they induced apoptotic characters such as morphology change, accumulation of cells in sub-G1 phase, and Annexin V positive expression. These effects together with the ability of roe protein hydrolyzates to induce reactive oxygen species and superoxide generation and mitochondrial depolarization make it a promising therapy against oral cancer.

